# Supplementary material for: Immune Profile of Blood, Tissue and Peritoneal Fluid: A Comparative Study in High Grade Serous Epithelial Ovarian Cancer Patients at Interval Debulking Surgery
Source: Vaccines (Basel). 2022 Dec 12;10(12):2121. doi: 10.3390/vaccines10122121 (PMC9784879; doi:10.3390/vaccines10122121)
Supplement: Supplementary file 1 [file vaccines-10-02121-s001.zip › Supplementary Figure S2 03112022.pdf]

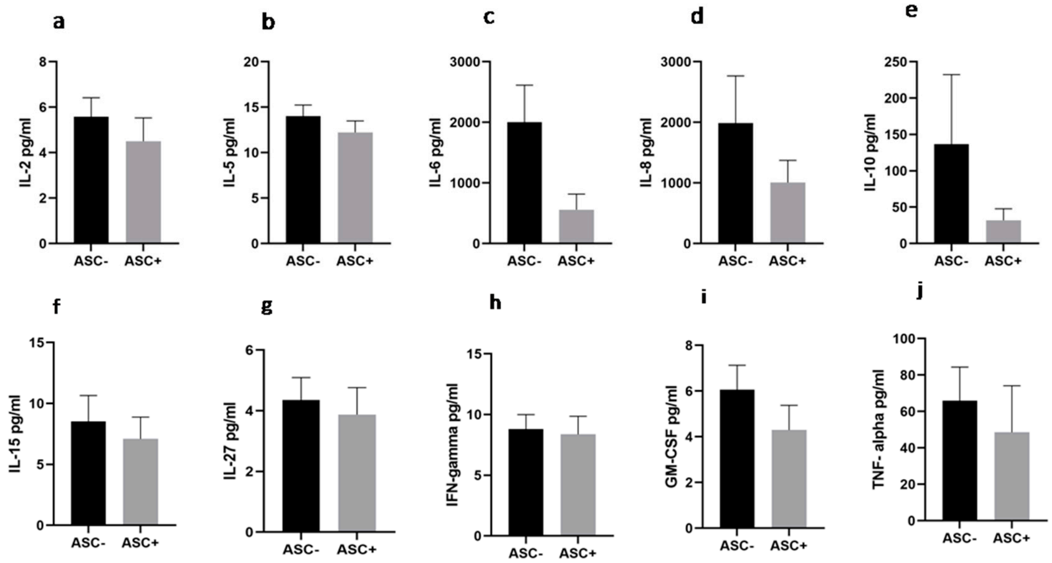

Supplementary Figure S2 Cytokine profile in fluid cytology positive (ASC+) and fluid cytology negative (ASC-) peritoneal fluid (PF) sample of HGSOC patients
