# Supplementary material for: Immune Profile of Blood, Tissue and Peritoneal Fluid: A Comparative Study in High Grade Serous Epithelial Ovarian Cancer Patients at Interval Debulking Surgery
Source: Vaccines (Basel). 2022 Dec 12;10(12):2121. doi: 10.3390/vaccines10122121 (PMC9784879; doi:10.3390/vaccines10122121)
Supplement: Supplementary file 1 [file vaccines-10-02121-s001.zip › Supplementary Table S1 03112022.pdf]

Supplementary Table S1 Details of antibodies used in the study

Panel for NK cell receptors			
Panel 1<br>Fluorescent labeled antibody	Clone	Panel 2<br>Fluorescent labeled antibody	Clone
Vivid dye- BV421		Vivid-Dye-BV421	
CD45-PECY5.5	HI30	CD45-PECY5.5	HI30
CD56-PE	5.1H11	CD56-PE	5.1H11
CD3-PECF594	UCHT1	CD3-PECF594	UCHT1
NKG2D-APC-CY7	1D11	NKp44-PECY7	P44-8
CD161-BV480	DX12	NKG2C-AF488	134522
DNAM-1-BV786	11A8	KIR2DL1/S1-APC	EB6B
NKp30-BV605	p30-15	KIR3DL1-BV786	DX9
NKp46-PerCP-cy5.5	9E2		
NKG2A-APC	131411		
KIR2DL2/L3/S3-AF488	180704		
